# Temporal bone carcinoma with intracranial extension

**DOI:** 10.1016/S1808-8694(15)30531-0

**Published:** 2015-10-18

**Authors:** Shitij Arora, J K Sharma, Sunil Pippal, Yatin Sethi, Abhinav Yadav

**Affiliations:** 1 DR (resident ENT, gandhi medial college bhopal india); 2 DR (prof & head, deptt of ent, gandhi medical college, bhopal india); 3 DR (prof & head, deptt of ent, gandhi medical college, bhopal india); 4 DR (resident ENT, gandhi medial college bhopal india); 5 DR (resident ENT, gandhi medial college bhopal india)

**Keywords:** carcinoma, intracranial, temporal

## INTRODUCTION

Squamous Cell Carcinoma of the temporal bone is an uncommon entity accounting for fewer than 0.2% of all tumors of the head and neck and is associated with a poor outcome^1^. Temporal bone carcinoma includes cancers arising from pinna that spreads to the temporal bone, primary tumors of the external auditory canal (EAC), middle ear, mastoid, petrous apex and metastatic lesions to the temporal bone. Malignancies of the temporal bone arise most commonly from the pinna as it undergoes many years of sun exposure.

## CASE REPORT

A 57 year old patient presented at the ENT OPD of Hamidia Hospital, Bhopal with complaints of discharge from right ear since 6 months, right preauricular swelling since 15 days and right sided facial nerve palsy since 15 days. On otologic examination there was a diffuse bony hard swelling of about 5 × 5cm involving the right preauricular and zygomatic region. External auditory canal was completely obliterated by a fleshy polypoidal mass covered with discharge. Tuning fork tests revealed a moderate conductive hearing loss on right side.

**Figure 1:**
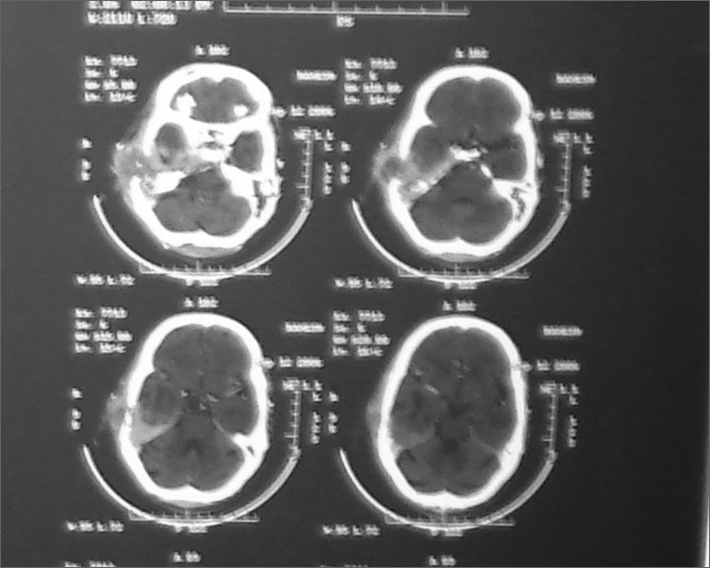
CT scan temporal bone showing the local destruction with intracranial extension

High resolution CT scan of temporal bone and head revealed a large soft tissue mass showed post contrast enhancement involving middle ear cavity and external auditory canal (fig 1). Extensions were into right masticator space, right parotid space with destruction of upper part of the pterygoid, mastoid, squamous, petrous part and clivus of temporal bone. Mass also extended into right temporal lobe with surrounding oedema and extensive soft tissue involvement.

Biopsy was taken from external auditory canal and revealed the mass as Squamous cell carcinoma (adenoid pattern). Owing to the poor cardiovascular and pulmonary status as well as unresectability surgery was deferred. Patient underwent high dose induction chemotherapy with Methotrexate(400mg/kg) and Mitomycin(10mg/sq m) followed by radiotherapy, dose of which was 7000 rads with brain exposure lessened by 1000 rads. Tumor shrinked and patient's facial palsy recovered however complete remission could not be achieved and patient succumbed to his disease within 3 months of presentation.

## DISCUSSION

Chronic otitis media and cholesteatoma are common in patients with temporal bone cancers and have been implicated as etiologic factors^5^. Human papilloma virus has been implicated in squamous cell carcinomas of the middle ear. Lim et al (2000) reported a series of temporal bone cancers in 7 patients who had undergone radiotherapy for nasopharyngeal carcinoma^4^. Nodal metastasis is uncommon in early disease but may occur in 10-20% of cases of advanced disease^5^. Distant metastasis is rare.

Specific radiographic information is crucial for accurate preoperative staging. A fine-cut (1 mm) high-resolution CT scan of the temporal bone should be obtained. MRI with gadolinium enhancement can be helpful as it delineates soft tissue interfaces. An audiogram is obtained prior to performing any major procedure on the ear or temporal bone. To date, no staging system for temporal bone malignancies is universally accepted^3^. A staging system for squamous cell cancers of the EAC proposed by the University of Pittsburgh has been shown useful and has gained support in the literature ^1^,^2^.

Primary radiation is ineffective as curative treatment. Postoperative radiation treatment may be indicated in advanced disease^4^. In general all patients who are medically fit should undergo surgical treatment with en bloc removal of all cancer.

The optimal management of temporal bone cancer remains unclear because of continued debate regarding staging, the utility of preoperative radiographic evaluation, the extent and nomenclature of surgical procedures and the use of adjuvant radiation. The limited number of cases of temporal bone malignancies at each individual institution precludes definitive conclusions regarding the optimum protocol for management.
